# Biological and economic responses to increasing nitrogen rates in Mombaça guinea grass pastures

**DOI:** 10.1038/s41598-022-05796-6

**Published:** 2022-02-04

**Authors:** Valéria Pacheco Batista Euclides, Denise Baptaglin Montagner, Alexandre Romeiro de Araújo, Mariana de Aragão Pereira, Gelson dos Santos Difante, Itânia Maria Medeiros de Araújo, Leandro Francisco Barbosa, Rodrigo Amorim Barbosa, Antonio Leandro Chaves Gurgel

**Affiliations:** 1 Embrapa Beef Cattle, Av. Radio Maia, 830, Campo Grande, MS 79106-550 Brazil; 2 Animal Science Post-Graduation Program, Federal University of Mato Grosso Do Sul, Av. Sen. Filinto Müler, 2443, Campo Grande, MS 79070-900 Brazil; 3 Animal Science Post-Graduation Program, Federal University of Grande Dourados, Rod.Dourados/Itahum, Km 12, C.P. 364, Dourados, MS 79.804-970 Brazil

**Keywords:** Plant sciences, Environmental sciences

## Abstract

Nitrogen fertilization has been recognized as an essential tool towards the establishment of sustainable intensification of pasture-based livestock systems using tropical perennial grasses if, for a given ecosystem it is capable of increasing forage growth, stocking rates and animal performance. This study assessed pasture growth traits, nutritive value, animal and economic responses of *Panicum maximum* cv. Mombaça guinea grass pastures subjected to different levels of N fertilization (100 (N100), 200 (N200), and 300 (N300) kg N ha^−1^ yr^−1^). Pastures were managed under rotational stocking to maintain similar pre (80–90 cm) and post-grazing (45 cm) canopy heights. A partial budget and a Benefit–Cost Analysis were used to assess the economic returns on increasing N fertilization. N300 resulted in greater post-grazing herbage mass. A slightly higher neutral fiber and acid lignin detergent was observed at N100 (*P* < 0.05); crude protein increased linearly, and in vitro digestible organic matter reached maximum value at 265.4 kg N ha^−1^ yr^−1^. Annual averages of animal weight gain were 515, 590 and 660 g d^−1^, respectively, for N100, N200 and N300. There was a decrease from 3.7 to 1.9 kg of body weight gain per kg of additional N applied when increasing N rates from 100 to 200 and from 100 to 300 kg ha^−1^. The net profit improved with increasing N levels, but at reducing rates, reaching its maximum at the N300 level. The change from 100 to 200 kg N ha^−1^ presented the best return, with USD 3.73 for each additional dollar invested, while the change from 200 kg N ha^−1^ to 300 kg N ha^−1^ was economically less than optimal, recouping only USD 1.60 for each dollar. The N300 rate presented the highest net profit per hectare (accounting profit), even in a pessimist scenario (25% reduction in production). Despite being profitable, the N300 rate was less than optimal from an economic standpoint, since an additional 100 kg of Nitrogen ha^−1^ to change from N200 to N300 level reduced both the net returns and the Benefit–Cost ratio. Our results suggest that the economically optimal level of N fertilization for Mombaça guinea grass pasture should be between 200 and 300 kg ha^−1^.

## Introduction

In Cerrado regions of Brazil, it is estimated that there are at least 32 million ha of degraded pastures^1^, i.e., areas characterized by a decrease in regrowth vigor, consequently reducing carrying capacity and animal production, which results in great economic and environmental damages. Nitrogen fertilization is essential for maintaining pasture productivity and for its sustainability, since nitrogen deficiency is one of the main factors triggering pasture degradation process^2,3^.

Herbage productivity and stocking rates have been shown to increase as result of N-fertilizer inputs in pasture-based systems^4–6^; however there is an economic limit to N fertilizer N use, because of its elevated cost. Excessive N application has also been associated with an increase in greenhouse gas (GHG) emissions with consequences for environmental quality^7^. Grazing management strategies that optimize herbage utilization and digestible dry matter intake could mitigate environmental issues of pasture-based cattle systems. In this context, Congio et al*.*^8^ showed that it is possible to reduce 40% in the N-N_2_O emission intensity when appropriate grazing management is used.

Studies involving tropical pastures, particularly those using rotational grazing methods, have shown that the point at which the canopy intercepts 95% of photosynthetically active radiation should be considered the ideal period to interrupt regrowth. After this point, forage accumulation decreases^9–12^ and undesirable changes occur in sward canopy structure, such as increases in the stalk and senescent material fractions. In this context, using this target for *Panicum maximum* cv. Mombaça resulted in a greater proportion of leaves and a lower proportion of stem in the canopy, as well as greater forage nutritive value, when compared to pastures managed with 100% interception^13^. Another important point is identifying the optimal time for interruption of the grazing process to ensure higher forage intake rates and consequently better animal performance. Euclides et al*.*^15^ observed that sward structure, nutritive value, herbage intake and animal performance were affected by post-grazing residue height, and an increase of 40% in body weight gain per animal and per area was obtained when Mombaça guinea grass pastures was rotationally stocked to leave a 50-cm vs. a 30-cm post-grazing residue height, when gazing started at 95% canopy light interception.

Additionally, much uncertainty exists about the best way to adjust fertilizer rates for different grass species and soil conditions, considering that bioeconomic efficiency of N fertilization depends on the efficiency of N conversion to forage, grazing efficiency and conversion of forage consumed to animal product^16^. However, there is a knowledge gap in the relationship between plant and animal responses with increasing N fertilization rates in Mombaça guinea grass pasture.

Our hypothesis was that, in association with adequate grazing management, N fertilization is a tool for establishment of sustainable intensification of pasture-based livestock systems using Mombaça guinea grass. Our objective was to investigate the effect of increasing N-rates on plant traits, animal production, and economic response in Mombaça guinea grass pastures in the Brazilian Cerrado (Table 1).

**Table 1:** Chemical characteristics (0–10 cm and 0–20 cm) throughout the experimental period.

Chemical characteristics	2014						2017					
	100 N		200 N		300 N		100 N		200 N		300 N	
	0–10	0–20	0–10	0–20	0–10	0–20	0–10	0–20	0–10	0–20	0–10	0–20
pH–CaCl_2_	5.89	6.04	5.82	5.94	5.89	5.96	5.37	5.63	5.43	5.60	5.33	5.53
Ca^++^ (cmolc dm^−3^)	4.40	4.32	4.10	3.85	4.17	4.08	2.58	2.37	2.53	2.60	2.26	2.24
Mg^++^ (cmolc dm^−3^)	1.18	1.12	1.12	1.05	1.08	1.12	1.37	1.34	1.28	1.25	1.16	1.14
H + Al (cmolc dm^−3^)	3.92	3.51	3.48	3.29	3.70	3.77	3.30	2.84	2.95	3.10	4.28	3.37
S (cmolc dm^−3^)	5.94	5.81	5.48	5.20	5.85	5.61	4.34	4.04	4.15	4.11	3.80	3.67
T (cmolc dm^−3^)	9.86	9.32	8.96	8.49	9.55	9.38	7.64	6.88	7.10	7.21	8.08	7.04
V (%)	60.24	62.33	61.16	61.24	61.25	59.80	56.90	58.58	58.45	57.03	47.29	52.41
m (%)	0.00	0.00	0.00	0.00	0.00	0.00	0.32	0.35	0.41	0.25	0.45	0.37
OM (%)	4.10	4.03	3.89	3.39	4.29	4.24	4.49	4.03	3.79	3.79	4.33	3.90
P-Mehlich 1 (mg dm^−3^)	3.98	2.32	6.62	2.94	5.31	2.81	6.52	3.89	10.09	6.49	8.68	3.66
K^+^-Mehlich 1 (mg dm^−3^)	140.8	148.6	101.7	117.3	234.6	160.3	156.4	129.0	132.9	101.7	152.5	113.4

S = bases sum; T = cation exchange capacity (pH7); V = base saturation; m = aluminum saturation; OM = organic matter.

## Results

The pre-grazing canopy heights of Mombaça guinea grass pastures remained within the target range during the rainy seasons (Table 2). However, during the dry period, in order to maintain the target heights, some pastures required destocking (Seal the pasture); even so, these pasture’s pre-grazing canopy heights were below the targeted level (Table 2). The post-grazing target heights were maintained close to the target value of 45 cm during both seasons (Table 2).

**Table 2:** Average and standard deviation (±) for the pre- and post-grazing sward heights of *P. maximum* cv. Mombaça guinea grass pastures subjected to rotational stocking and fertilized with three nitrogen levels and the period which pasture was deferred, throughout the experiment.

Season	kg N ha^−1^					
	N100		N200		N300	
	Pre (cm)	Post (cm)	Pre (cm)	Post (cm)	Pre (cm)	Post (cm)
**2014–2015**						
Rainy	82.4 ± 1.8	44.6 ± 0.9	83.4 ± 2.0	43.8 ± 0.8	85.1 ± 2.0	45.4 ± 0.8
Dry	63.1 ± 4.5	44.8 ± 3.4	69.7 ± 2.3	44.1 ± 1.4	75.3 ± 4.0	45.2 ± 1.3
Deferred (days)	25		13		–	
**2015–2016**						
Rainy	84.5 ± 2.5	47.5 ± 1.1	85.4 ± 1.5	47.1 ± 1.6	85.7 ± 1.7	48.0 ± 1.1
Dry	65.0 ± 7.2	42.8 ± 2.4	63.9 ± 4.1	43.8 ± 1.8	72.0 ± 5.7	45.4 ± 2.3
Deferred (days)	33		26		15	
**2016–2017**						
Rainy	82.8 ± 1.2	45.9 ± 1.0	83.2 ± 1.4	46.0 ± 1.5	83.8 ± 1.4	47.1 ± 0.9
Dry	67.8 ± 3.4	43.8 ± 1.8	67.5 ± 3.9	42.3 ± 1.5	69.8 ± 2.9	45.8 ± 1.7
Deferred (days)	61		35		21	

An interaction between the effect of N rate and season was observed for rest and grazing (*P* < 0.05) periods. During the rainy season, pastures fertilized with 100 kg N ha^−1^ (N100) required longer rest period (*P* < 0.05) than those fertilized with 200 kg N ha^−1^ (N200), which in turn had greater (*P* < 0.05) rest periods than pastures fertilized with 300 kg N ha^−1^ (N300). In the dry season, rest period was greater (*P* < 0.05) for pastures fertilized with N100 than pastures fertilized with N200 and N300. Regardless the N rate, the length of rest period was lower (*P* < 0.05) during the rainy than in dry season (Table 3).

**Table 3:** Averages and standard error of mean (±) for rest and grazing periods of *P. maximum* cv. Mombaça guinea grass pastures subjected to rotational stocking and fertilized with three nitrogen levels, during the rainy and dry season.

Season	kg N ha^−1^		
	N100	N200	N300
**Rest period (days)**			
Rainy	43Ba ± 2.3	35Bb ± 2.0	29Bc ± 2.0
Dry	67Aa ± 2.9	46Ab ± 2.5	41Ab ± 2.3
**Grazing period (days)**			
Rainy	6.4Aa ± 0.17	5.7Bb ± 0.14	4.7Bc ± 0.13
Dry	7.0Aa ± 0.24	6.4Aab ± 0.18	5.9Ab ± 0.17

Values followed by different letters, lower case in row and upper case in column, were significantly different according to the Tukey test at *P* < 0.05.

During the rainy season, the grazing period was longer (*P* < 0.05) for pastures fertilized with N100 than those fertilized with N200, which in turn were longer (*P* < 0.05) than pastures fertilized with N300. During the dry season, pastures fertilized with N100 had longer (*P* < 0.05) grazing periods compared with N300, while pasture with N200 was intermediate with similar (*P* > 0 0.05) grazing period length to the other N rates. Except for pasture fertilized with N100, the length of grazing period was greater (*P* < 0.05) in the dry than in the rainy season (Table 3).

There were no interactions (*P* > 0.05) among N rate, season and experimental year; N rate × season; N rate × experimental year; and season × experimental year, for the other variables studied.

**Figure 1:**
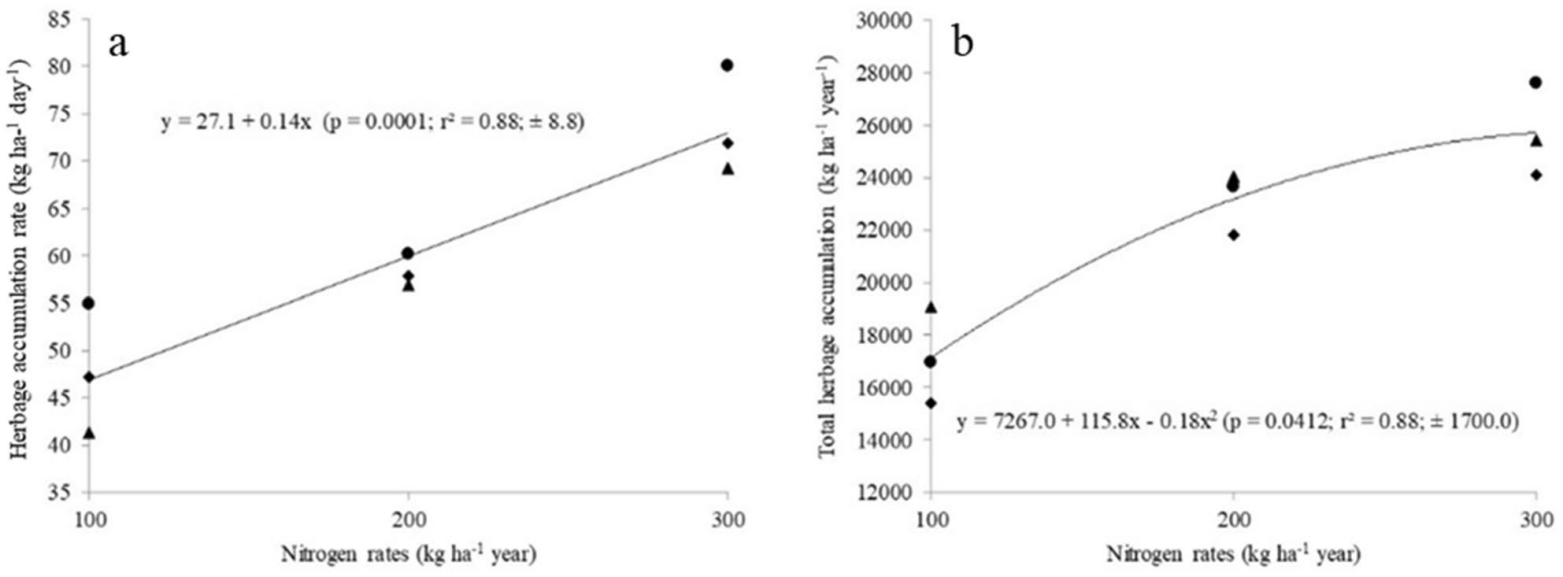
Nitrogen fertilization effect on herbage accumulation rate (**a**), and herbage accumulation per year (**b**) in *P. maximum* cv. Mombaça guinea grass pastures subjected to rotational stocking and nitrogen rates (● year 1; ♦ year 2; ▲ year 3).

**Figure 2:**
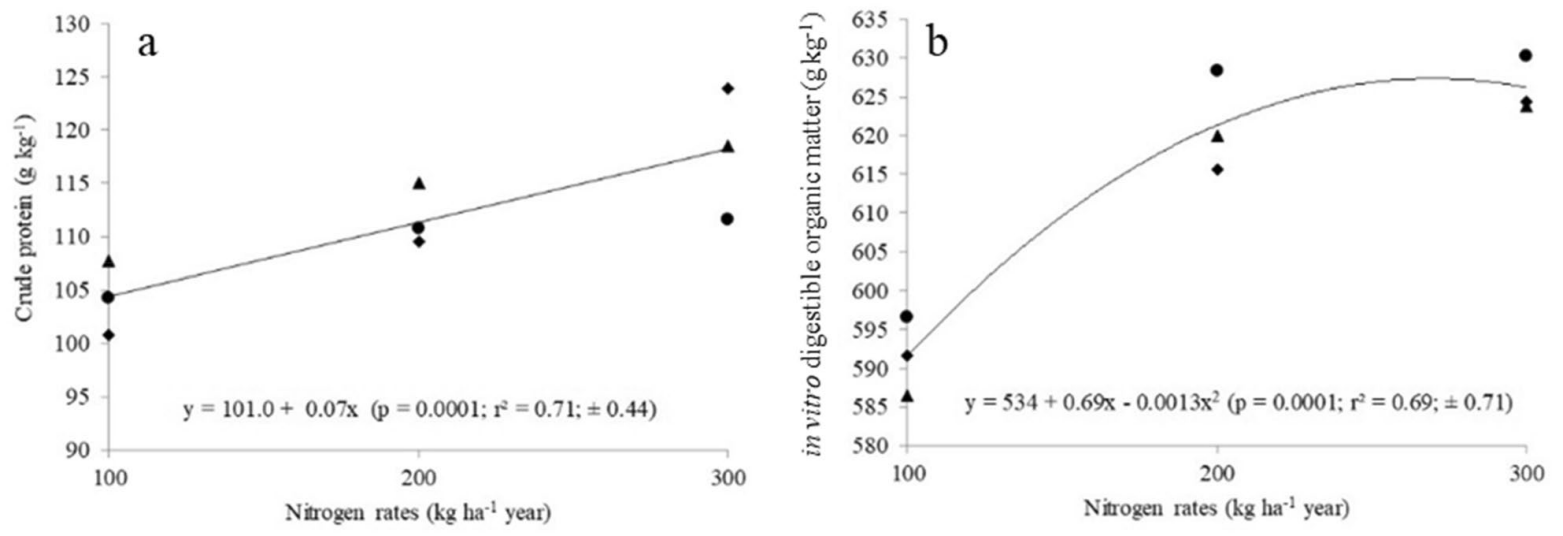
Effect of nitrogen on crude protein (**a**) concentration and in vitro digestible organic matter (**b**) percentage of leaves of *P. maximum* cv. Mombaça guinea grass pastures subjected to rotational stocking and nitrogen rates (● year 1; ♦ year 2; ▲ year 3).

**Figure 3:**
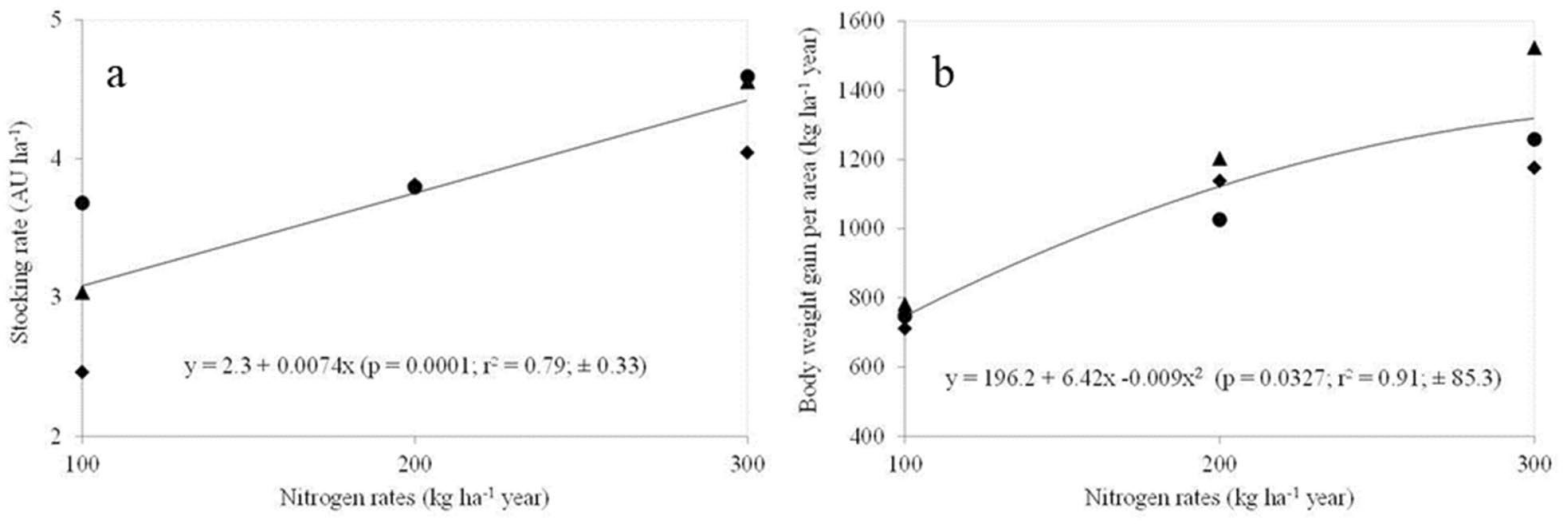
Effect of nitrogen on stocking rate (**a**) and body weight gain per area (**b**) in *P. maximum* cv. Mombaça guinea grass pastures subjected to rotational stocking and nitrogen rates (● year 1; ♦ year 2; ▲ year 3); (AU: Animals of 450 kg of body weight).

There was a linear effect (*P* < 0.05) of N fertilization for herbage accumulation rate (HAR), crude protein (CP) and stocking rate (SR). The HAR (Fig. 1a), CP concentration (Fig. 2a) and SR (Fig. 3a) increased (*P* < 0.05) with increasing N rates. Total herbage accumulation (THA), body weight gain per area (BWGA) and in vitro digestible organic matter (IVDOM) were best fitted a second-order polynomial equation (*P* < 0.05). The THA (Fig. 1b), IVDOM percentage (Fig. 2b) and BWGA (Fig. 3b) increased at decreasing rate with increasing N rate (*P* < 0.05).

The linear model was not adequate (*P* > 0.05) for predicting average daily gain (ADG; r^2^ = 0.43), neutral detergent fiber (NDF; r^2^ = 0.28) and acid detergent lignin (ADL; r^2^ = 0.37) concentrations, since it showed low correlation coefficients. However, when N fertilization rate was considered as class variable, NDF and ADL were greater (*P* < 0.05) for pastures fertilized with N100 (748 and 32 g kg^−1^) than for pastures fertilized with N200 (741 and 29 g kg^−1^) and N300 (740 and 29 g kg^−1^), which did not differ (*P* < 0.05). The ADG was greater for pastures fertilized with N300 (0.660 kg steer^−1^) than those fertilized with N200 (0.590 kg steer^−1^) which in turn were greater than pastures with N100 (0.515 kg steer^−1^) (*P* < 0.05).

Pre-grazing herbage mass averaged 5.2 ± 0.09 t ha^−1^ of dry matter and was not affected by N rates (*P* > 0.05). Also, no effect of N rates was found for pre-grazing percentages of leaf, stem and dead material or leaf:stem ratio (*P* > 0.05). The mean (± standard error) values for the above variables were: 56.9 ± 1.3%; 17.3 ± 0.8%; 25.8 ± 1.7%; and 3.7 ± 0.2, respectively.

Post-grazing herbage mass was greater (*P* < 0.05) in N300 compared with N200 or N100, which did not differ. The mean (± standard error) values were: 3.2 ± 0.07; 3.0 ± 0.07; 2.9 ± 0.08, respectively. However, no effect of N rates was found for post-grazing percentages of leaf, stem and dead material (*P* > 0.05). The mean (± standard error) values for the above variables were: 19.9 ± 0.9%; 22.8 ± 1.2%; and 58.8 ± 1.5%, respectively.

Except for pre-grazing stem percentage and post-grazing herbage mass (*P* > 0.05), a season effect was observed (*P* < 0.05) for the other variables (Table 4). The HAR, stocking rate, ADG, pre-grazing herbage mass, leaf percentage, leaf:stem ratio, CP and IVDOM percentage were greater in the rainy season than during the dry season. The reverse was observed for dead material percentage, NDF and acid ADL concentrations. Leaf and stem percentages were greater and dead material percentage was lower post-grazing during the rainy season than during the dry season (*P* < 0.05) (Table 4).

**Table 4:** Average, standard error of mean (±)for herbage accumulation rate (HAR), stocking rate, average daily gain (ADG), pre-grazing herbage mass, leaf and dead material percentages, leaf:stem ratio, crude protein, neutral detergent fiber and acid detergent lignin concentrations, in vitro digestible organic matter (IVDOM) of leaves, and post grazing leaf, stem and dead material percentages in *P. maximum* cv. Mombaça guinea grass pastures subjected to rotational stocking and fertilized with three nitrogen levels, during the rainy and dry seasons.

	Season		*P* value
	Rainy	Dry	
**Variable**			
HAR (kg ha^−1^ day)	85.8 ± 1.8	31.4 ± 2.1	0.0001
Stocking rate (AU ha^−1^)	5.2 ± 0.2	1.6 ± 0.4	0.0001
ADG (kg steer^−1^)	0.730 ± 0.013	0.370 ± 0.016	0.0001
**Pre-grazing**			
Herbage mass (kg ha^−1^)	5,730 ± 53	4,749 ± 81	0.0001
Leaf (%)	65.7 ± 0.8	48.2 ± 1.2	0.0001
Dead material (%)	17.0 ± 1.0	34.5 ± 1.6	0.0001
Leaf:stem ratio	4.2 ± 1.1	3.1 ± 0.2	0.0001
Crude protein (g kg^−1^)	121 ± 1.0	97 ± 2.0	0.0001
Neutral detergent fiber (g kg^−1^)	736 ± 2.0	749 ± 1.0	0.0001
Acid detergent lignin (g kg^−1^)	29 ± 0.3	31 ± 0.4	0.0001
IVDOM (g kg^−1^)	630 ± 2.0	588 ± 3.0	0.0001
**Post-grazing**			
Leaf (%)	22.3 ± 0.6	17.4 ± 0.8	0.0001
Stem (%)	28.0 ± 0.8	17.5 ± 1.0	0.0001
Dead material (%)	49.9 ± 1.0	66.0 ± 1.3	0.0001

There was no year effect for pre-grazing herbage mass, stem percentage and CP (*P* > 0.05). However, HAR, SR and the body BWGA were similar (*P* > 0.05) in the second and third years and lower than what was observed in the first year (*P* < 0.05). The inverse was observed for the ADG, which was similar (*P* > 0.05) in the first and second years and lower (*P* < 0.05) than the third one (Table 5).

**Table 5:** Average and standard error of mean (±) and probability level (*p*) for herbage accumulation rate (HAR), stocking rate, average daily gain (ADG), body weight gain per area (BWGA), pre-grazing leaf and dead material percentages, leaf:stem ratio, neutral detergent fiber and acid detergent lignin concentrations, in vitro digestible organic matter (IVDOM) percentage of leaves, post-grazing herbage mass, leaf, stem and dead material percentages for *P. maximum* cv. Mombaça guinea grass pastures subjected to rotational stocking and fertilized with three nitrogen levels, in the experimental years.

	Years			*P* value
	2014/2015	2015/2016	2016/2017	
**Variables**				
HAR (kg ha^−1^ day)	64.6a ± 2.1	57.1b ± 1.9	54.1b ± 2.0	0.0055
Stocking rate (AU ha^−1^)	4.0a ± 0.13	3.4b ± 0.15	3.1b ± 0.16	0.0002
ADG (kg steer^−1^)	0.545b ± 0.017	0.510b ± 0.018	0.635a ± 0.019	0.0006
BWGA (kg ha^−1^ year)	1180a ± 32	1005b ± 32	1115ab ± 36	0.0083
**Pre-grazing**				
Leaf (%)	57.4a ± 1.5	52.9b ± 1.1	60.4a ± 1.0	0.0001
Dead material (%)	26.0ab ± 2.0	29.1a ± 1.4	22.2b ± 1.3	0.0053
Leaf:stem ratio	3.9a ± 0.3	3.1b ± 0.2	4.0a ± 0.2	0.0053
Neutral detergent fiber (g kg^−1^)	735b ± 2.4	748a ± 2.1	747a ± 1.8	0.0001
Acid detergent lignin (g kg^−1^)	28b ± 0.6	31a ± 0.5	31a ± 0.4	0.0001
IVDOM (g kg^−1^)	617a ± 3.1	605b ± 2.8	606b ± 2.4	0.0070
**Post-grazing**				
Herbage mass (kg ha^−1^)	2959ab ± 113	3192a ± 71	2879b ± 70	0.0062
Leaf (%)	21.6a ± 1.0	18.5b ± 0.9	19.5ab ± 0.8	0.0446
Stem (%)	25.4a ± 1.2	20.0b ± 1.1	22.9ab ± 1.1	0.0077
Dead material (%)	53.4b ± 1.6	61.6a ± 1.4	58.9a ± 1.4	0.0007

Values followed by different letters in row, were significantly different according to the Tukey test at *P* < 0.05.

Pre-grazing leaf percentage and leaf:stem ratio observed for the first and third years was greater than that for second year (*P* < 0.05). Dead material percentage was greater in the second year than that in the third one (*P* < 0.05), while in the first year showed a similar (*P* > 0.05) value as the other years (Table 5).

The NDF and ADL concentrations were similar during the second and third years (*P* > 0.05) and greater than that observed in the first year (*P* < 0.05). The inverse was observed for IVDOM (Table 5).

The post-grazing herbage mass was greater in the second year than that in the third one (*P* < 0.05), while during the first year it was similar to the others (*P* > 0.05). The leaf and stem percentages were greater in the first year when compared to the second (*P* < 0.05), while in the third year showed a similar value as the other years (*P* > 0.05). The dead material percentage was similar during the second and third years (*P* > 0.05) and greater than that observed in the first year (*P* < 0.05) (Table 5).

Considering the enterprise budget (i.e., pasture-based beef) for establishing and managing one hectare of Mombaça cultivar at increasing N-fertilizer rates, the net profit improved with increasing N rates (Table 6), achieving its highest level with N300 (USD 632.24 ha^−1^). When a partial budget was considered (Table 7), focusing on the net changes in costs and income in paired treatments, the change from N100 to N200 treatment showed the best Benefit–Cost Ratio (BCR). For every additional dollar invested to reach the N200 level, the system returned additional USD 3.73, while the change from N200 to N300 returned only USD 1.60. This means that any additional production at N300 level is done at a higher relative cost, as evidenced by the 48% reduction in benefits and 21% increase in variable input costs, when the effect of additional 100 kg of Nitrogen ha^−1^ are compared between paired treatments (Table 8).

**Table 6:** Enterprise budget for implementing one hectare of *P. maximum* cv. Mombaça guinea grass at different N-fertilizer levels for pasture maintenance.

	N rate (kg ha^−1^)		
	100 N	200 N	300 N
**Gross income (USD)**^2^			
Gross production (kg LW/ha)	1,386.0	1,701.0	2,025.0
Price (USD/kg LW)	1.39	1.39	1.39
Gross income (USD)	1,932.80	2,372.07	2,823.90
**Input costs (USD)**			
Total fixed input costs^3^	1,435.90	1,435.90	1,435.90
Variable costs (fertilization)	318.41	427.60	564.44
Variable costs (cattle maintenance)	130.95	160.71	191.32
Total input costs	1,885.26	2,024.20	2,191.66
**Net profit (USD/ha)**	47.54	347.87	632.24

^1^Currency exchange 1.0 BRL = 0.258 USD (2018/2019 average, based on 26 observations over this period. Source: https://www.xe.com/currencyconverter/). ^2^According to FAO (2020), the value of gross income is obtained by multiplying “the gross production, in physical terms, by the output price at the farm gate”. In this table, the gross production is the three-year average (experimental period). ^3^ Includes cattle purchase.

**Table 7:** Partial budget for beef production on Mombaça guinea grass at increasing N-fertilizer rates^1^, based on experimental data and in an alternative scenario (25% reduction in yield).

	N rate (kg ha^−1^)			N rate (kg ha^−1^) 25% lower yield		
	100 N	200 N	300 N	100 N	200 N	300 N
**Gross income**						
Average yield (kg LW/ha)	748.0	1,120.0	1,312.0	561.0	840.0	984.0
Price (USD/kg LW)	1.39	1.39	1.39	1.39	1.39	1.39
Gross income (USD)	1,043.10	1,561.86	1,829.61	782.32	1,171.39	1,372.20
**Variable Input costs**						
Fertilization costs (USD)	318.41	427.60	564.44	318.41	427.60	564.44
Herd costs (USD)	130.95	160.71	191.32	130.95	160.71	191.32
Total input costs (USD)	449.36	588.31	755.76	449.36	588.31	755.76
**Net profit (USD)**	593.74	973.55	1,073.85	332.96	583.09	616.44

Net changes	Change (USD)		Change (USD) 25% lower yield			
	100–200	200–300	100–200	200–300		
Change in benefits between consecutive treatments (USD)^a^	518.76	267.75	389.07	200.81		
Change in total variable input costs between consecutive treatments (USD) ^b^	138.95	167.45	138.95	167.45		
Net returns	379.81	100.30	250.12	33.36		
Benefit–Cost Ratio (BCR)	3.73	1.60	2.80	1.20		

^1^Currency exchange 1.0 BRL = 0.258 USD (2018/2019 average, based on 26 observations over this period. Source: https://www.xe.com/currencyconverter/). ^a^Change in gross income (benefits), when comparing N200 to N100, and N300 to N200, in this order. ^b^Change in total variable costs, when comparing N200 to N100, and N300 to N200, in this order.

**Table 8:** Partial budget for beef production on Mombaça guinea grass at increasing N-fertilizer rates in an alternative scenario (25% reduction in yield).

	N rate (kg ha^−1^)			Change (%)	
	100 N	200 N	300 N	100–200	200–300
**Gross income (USD)**					
Average yield (kg LW/ha)	561.0	840.0	984.0	50%	17%
Price (USD/kg LW)	1.39	1.39	1.39	0%	0%
Gross income (USD)	782.32	1,171.39	1,372.20	50%	17%
**Input costs (USD)**					
Variable costs -fertilization	318.41	427.60	564.44	34%	32%
Variable costs—cattle	130.95	160.71	191.32	23%	19%
Total input costs	449.36	588.31	755.76	31%	28%
**Net benefit**	332.96	583.09	616.44	75%	6%

Net changes	Change (USD)		Difference (%)		
	100–200	200–300			
Change in benefit between consecutive treatments (USD)^a^	389.07	200.81	−48.4%		
Change in total variable input costs between consecutive treatments (USD) ^b^	138.95	167.45	21%		
Benefit–Cost Ratio (BCR)	2.80	1.20	−57.1%		

^1^Currency exchange 1.0 BRL = 0.258 USD (2018/2019 average, based on 26 observations over this period. Source: https://www.xe.com/currencyconverter/). ^a^Change in gross income (benefits), when comparing N200 to N100, and N300 to N200, in this order. ^b^Change in total variable costs, when comparing N200 to N100, and N300 to N200, in this order.

In an alternative scenario, where costs remained equal, but production reduced 25% across all treatments, the economic performance worsened, as expected (Table 8). Despite remaining *financially* viable, given the positive net profits for all N rates (i.e., positive cash flow), the BCR for N300 dropped to 1.2, indicating a deterioration of the investment capital and its ability of generating further value to the enterprise. While investing one dollar to reach the N200 level would return additional 2.8 USD in this scenario, the N300 would recoup only 1.2 USD per invested dollar, which indicates the N300 is a less-than-optimal alternative, from an economic standpoint.

## Discussion

### Nitrogen effect

Increased herbage accumulation rates (Fig. 1a) are similar to other studies involving tropical pastures^6,17,18^. The positive N fertilization effect could be explained by the fact that N fertilization accelerates growth^6,19–21^, tillering^6,22–24^, leaf appearance^22^, consequently, the expansion of the aerial part^20,25^. N is the nutrient that results in the greatest effect on the productivity of forage plants, as long as the other production factors are not limiting. Forage production is a function of morphogenic characteristics such as leaf appearance and elongation rate and leaf life span, depending on genotype and environmental factors, including N availability^26^.

The pre-grazing canopy height was Similar (Table 2) for all N rate; thereby, as the dose of N increased the pastures needed fewer days to reach the pre-grazing target height and ultimately increased the number of grazing cycles (averaging 6.3; 8.0 and 9.7, respectively for N100, N200 and N300). Consequently, the total annual HAR increased, but the magnitude of the increase decreased as N rate increased from N100 to N300 (Fig. 1b). The N-use efficiencies were: 173; 119 and 89 kg of dry matter per kg N applied, respectively, for the pastures fertilized with N100, N200 and N300. Reports in the literature show that, when adding increasing doses of nitrogen in tropical forages, the greatest increase in production is obtained with the first dose. With successive applications of the nutrient, production increments are increasingly smaller^27,28^. This phenomenon can be explained by the law of decreasing increments, which says that when adding increasing doses of a nutrient, the greatest increase in production is obtained with the first dose.

As a consequence of grazing management to control canopy height targets, the stocking rate (Fig. 3a) showed the same pattern of variation described for HAR (Fig. 1a), i.e. with increasing the N levels a higher stocking rate was required in order to achieve the pre- and post-grazing canopy height targets. The latter corroborates the observations reported by Hernandez Garay et al*.*^29^ who suggested that in order to achieve an efficient conversion of herbage production into meat, the stocking rate must be increased in function of herbage production caused by higher N rates. These results were in line with N-responses of the tropical grasses^6,30^^,^^31^.

Since the pastures were managed based on target pre-grazing canopy heights between 80 and 90 cm, the pre-grazing herbage mass and the morphological composition of the forage were similar for pastures with different N rates. These similarities were probably due to canopies were intercepting similar proportions of incident radiation at the end of the rest period. In this context, Barbosa et al*.*^10^, Zanine et al*.*^32^ and Sbrissia et al*.*^33^ suggested that the height at which the canopy intercepts 95% LI could be considered the optimum maximum height for interrupting regrowth when the objective is to maximize the productive capacity of pastures.

Positive effect of N rate on CP concentration (Fig. 2a) and IVDOM percentage (Fig. 2b) and decreased in neutral detergent fiber and acid detergent lignin for pasture fertilized with the highest N dose may be associated to a reduction in the average maturity of the herbage mass (Table 3) with increasing N. Similar results for tropical pastures were observed by, Dupas et al*.*^34^ and Delavatti et al.^6^. The increase in N rates also increased ADG most likely reflected the greater nutritive value of these pastures, since there were no differences in canopy structure to limit herbage intake.

Despite increases in SR (Fig. 3a) and ADG as N dose increased, the quadratic effect occurred for body weight gain per area (Fig. 3b). This fact means a decreased in the efficiency of N-use (gain in body weight per additional kg N applied), since the bodyweight gain per hectare increased by 372 kg ha^−1^ when N application increased from 100 to 200 kg ha^−1^, and that bodyweight gain per hectare increased by 192 kg ha^−1^ when N application increased from 100 to 300 kg ha^−1^. Such responses were consistent with those observed by Canto et al*.*^28^ in guinea grass pastures with N (100 to 400 kg N ha^−1^), which the N-use efficiency ranged from 3.9 to 2.1 kg BW gain per kg N; and by Delevatti et al*.*^6^ in palisade grass pastures N (0 to 270 kg N ha^−1^), which the N-use efficiency ranged from 2.8 to 1.7 kg BW gain per kg N. Martha Jr. et al*.*^3^ based on a review about efficiency of N-use in Brazilian pastures concluded that: an efficiency > 1.8 kg BW gain per kg N is considered good; and > 2.4 kg BW gain per kg N is considered excellent.

Fertilizers, in particular N, increase pasture growth rates (Fig. 1a), annual production (Fig. 1b) and nutritive value (Figs. 2a, b). However, pastures use the applied N fertilizer most effectively when other nutrients are in correct proportion to one another. Since P is often the most limiting nutrient for pasture production in highly weathered acidic soil of tropical America^35^, we must analyze if P fertilizations carried out over the years was appropriated of utilization of increasing N rates. It was observed along this trial increase of soil P contents (Table 1) as a function of fertilizer utilized (35 kg P ha^−1^ yearly). This P dose is very close to that proposed by Sousa et al*.*^36^ who suggested in this calculation to consider animal yield target (kg ha^−1^ body weight) and phosphorus requirement of forage species. Thus, regardless of the N rates, we consider the P replacement during pasture utilization to be adequate to keep stable animal production (Table 5). Potassium is the second element most absorbed by plants, and the amounts mobilized are a function of production. Accordingly, soil K extraction increased (Table 1) as herbage yield increase (Fig. 1b). We consider that the annual application of 66 kg K ha^−1^ was adequate^3^.

### Season effect

Regardless of N rate, the pre-grazing height was below the target during the dry season; however, N300 pastures showed greater pre-grazing height and less days without grazing than N200 and N100 (Table 2). Despite the adjustment of the stocking rate (Table 5) some paddocks needed to be deferred to maintain the post-grazing height targets (Table 2). The three years averages were: 40; 25 and 12 days without grazing, respectively for N100, N200 and N300 treatments.

**Figure 4:**
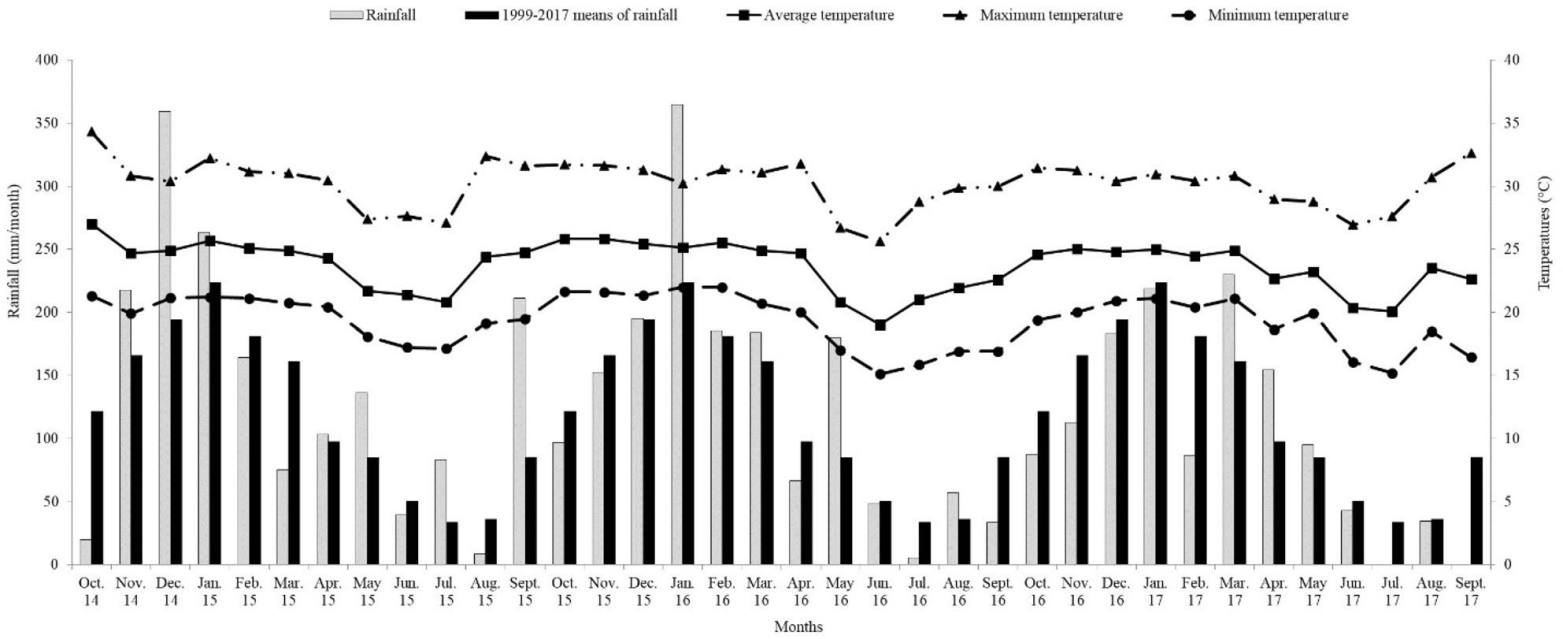
Monthly rainfall and minimum, average and maximum temperatures from September 2011 to July 2012, and historical 18-year (1999–2017) means of rainfall and medium temperature.

**Figure 5:**
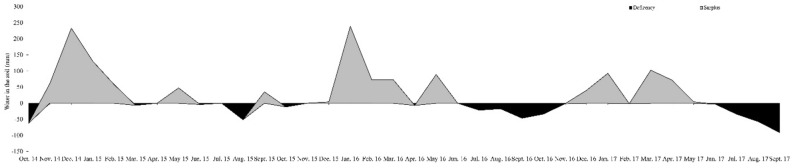
Monthly water deficit and surplus in the soil from September 2011 to July 2012, for a soil water holding capacity of 75 mm.

Regardless of the N rates the seasonal pattern of HAR (Table 4) is typical of tropical regions, resulting from rainfall seasonality (Figs. 4 and 5), in addition to temperature variations (Fig. 4) and photoperiod. A similar pattern in HAR for Mombaça guinea grass pastures was observed by Da Silva et al*.*^13^ and Galindo et al.^21^. As result of lower HAR, there were greater length of resting period (Table 3), lower pre-grazing canopy height (Table 2) and forage mass (Table 4). The decreased leaf and increased dead material (Table 4) percentages were related to the low leaf accumulation and natural senescence of grasses, which was accelerated by the water stress during the dry season (Fig. 5). Further, the reduction of CP and IVOMD percentage and increase NDF and ADL concentrations (Table 4) might be associated to increase in the average maturity of leaves in the herbage mass, during dry season, consequence of lower leaf appearance. Besides the lower nutritive value of the herbage and unfavorable canopy structure, i.e., lower leaf:stem ratio, for grazing may explain the lower animal performance observed during the dry (Table 4). Similar results for animal performance in Mombaça guinea grass pasture during the wet season was observed by Euclides et al*.*^37^, but greater than that obtained by Araújo et al*.*^38^ during the dry season.

### Year effect

Total rainfall recorded from October 2014 to September 2015 (1683 mm) and from October 2015 to September 2016 (1570 mm) was above 18-yr average (1450 mm), while it was bellow to the average from October 2016 to September 2017 (1245 mm). The 2015 dry season (479 mm) was wetter than the historical 18-year dry season (291 mm). On the other hand, dry season of 2016 (325 mm) was closed to the historical 18-yr average, while in 2017 (173 mm) shower drier weather from May to September (Figs. 4, 5). Soil water deficit was registered during for the entire dry season (May to September). The maximum and minimum average daily temperatures during experimental years was closed to the historical 18-yr maximum (30.1ºC) and minimum (19.0ºC) average daily tem*p*eratures.

The decrease in HAR and SR from the first to the second and third years (Table 5) was mainly related to climatic conditions, since in the first experimental year the total precipitation was higher than the other years of the experiment and was above the historical average. Furthermore, rainfall was better distributed in time and space (Fig. 4) compared to the second and third of the experiment. Furthermore, the soil water deficit, in the first experimental year, was recorded for only two months (July and August), while in the other years the soil water deficit was recorded for five months (Fig. 5). Considering that forage accumulation rate variation was direct affected by climate, the N fertilization shown to sustain the forage production and stocking rate, since they remained stable in the second and third years (Table 5).

On the other hand, the increase in body weight gain per area from the second to the third year could be explained by greater average daily gain observed during the third year (Table 5). This greater animal performance (2016–2017) most likely reflected the genetic group (½ Brahman- ½ Angus) of the animals utilized in that year, which has greater potential to gain body weight than the genetic group (½ Senepol × ½ Caracu) of the animals used during the first (2014–2015) and second (2015–2016) years. Such superiority is due to the hybrid vigor or heterosis, which is higher in crosses between Zebu and European breeds^39^.

### Economic response

Sustainable intensification of pasture ensuring farmers’ profitability is crucial for further adoption of fertilization practices, which, in turn, will result in higher beef supply. According to Molossi et al*.*^40^, the use of 100 kg ha^−1^ of N on *Brachiaria* pasture in the Brazilian Cerrado resulted in additional 82 kg carcass weight ha^−1^, which could feed 2.35 more people per hectare, considering the global projection for annual per capita consumption of meat of 35.1 kg person^−1^ in 2028, than extensive systems (with no fertilization). In the same way, our results suggest that increasing N rates from 100 kg ha^−1^ to 200 or 300 kg ha^−1^ could potentially feed 5.3 or 2.7 more people, respectively, per hectare.

The economic analyses carried out here, in general, showed the higher the N rate, the higher the total production level was and, consequently, the gross income (Table 6). After input costs were deducted, the same pattern was found for the net benefit, which increased across treatments, achieving its highest value at the N300 level. The recent work of Molossi et al*.*^40^, at some extent, corroborates our results. The authors, comparing farm-level profitability between extensive systems and systems with pasture N fertilization, found annual net returns of US$ 22.86 ha^−1^ and US$ 65.69 ha^−1^, respectively, for a Cerrado representative farm, and of US$ 24.28 ha^−1^ and US$ 66.75 ha^−1^, respectively, for an Amazon representative farm. Euclides et al*.*^41^, studying bioeconomic responses to maintenance N-fertilization on *P. maximum* cv. Tanzania, also found a better economic performance when N-fertilizer levels increased from 50 kg ha^−1^ to 100 kg ha^−1^. The additional net benefit per hectare exceeded the additional costs in 55%^41^. In both studies, however, N-fertilization levels were much lower (up to 100 kg ha^−1^ N) than the rates analyzed here, and comparisons may not be as straightforward, particularly for the N200 and N300 levels.

The partial budget analysis revealed a non-linear growth behavior between N-levels and profitability (Table 7). Net benefits grew rapidly from N100 to N200 (64%), reaching USD 380 per hectare due to the change, and, at a reduced rate, from N200 to N300 (10%), resulting in additional USD 100 per hectare. This is in accordance to the economic “law” of diminishing returns^42^, which poses that for every unit of a factor added (e.g., fertilizer), all else remaining constant, the return from additional levels of this factor will decrease until becomes negative. This is also evidenced by the benefit–cost ratio (BCR), where the change from N100 to N200 would provide farmers with additional USD 3.73, but the change from N200 to N300 would recoup only USD 1.60 per additional dollar invested. Regarding efficiency, the change from the application of 100 kg N ha^−1^ to 200 kg N ha^−1^ also allowed for greater N-use efficiency, with 3.7 kg of body weight per additional kg of N applied.

The findings by Soha^43^ had a similar pattern to our results, with N-fertilization increase from 100 to 200 to 300 kg of N ha^−1^ on sorghum resulting in growing net benefits, but reducing the Marginal Rate of Returns, leading to the conclusion that the N200 was the most profitable fertilization rate. Likewise, our results also indicate the N200 presented the best economic return (BCR = 3.73), whereas N300 proved to be less-than-optimal, in economic terms, despite having the highest net income of all treatments.

In an alternative scenario, where costs remained the same, but beef production reduced by 25%, the net profit dropped 44%, 40% and 43% for N100, N200 and N300, respectively, compared to the previous analysis (Table 8). Again, an investment of one dollar towards N200 level recouped the one dollar and gave an additional USD 2.80, while the N300 recouped just USD 1.20. According to Ratnatunga and Montali^44^, more important than accounting profit (i.e., cash-flow based) is to seek the improvement of long-term financial health of the business, which is granted by the returns on investment (operating investments, in our study). We, thus, suggest that profit-maximizing fertilization rates for Mombaça guinea grass, all else being constant, lies between 200 kg ha^−1^ and 300 kg ha^−1^ of N.

All results shown here hold true if the relative prices remain more or less stable. Major changes in this relationship will impact results accordingly. Pereira et al*.*^45^, claim that the economic performance is a function of input/output relative prices in integrated farming systems. Such a claim can be extended to any economic activity, including beef farming, and must be considered by farmers.

When making a decision with regards to levels of fertilization that improve profitability, farmers should properly evaluate any additional risk and capital requirements^46^. The rule of thumb is should the additional average profit outweighs the additional risks or variability of profits, then the new practice/technology should be adopted. In our study, the change from N200 to N300 rendered additional USD 100 profit ha^−1^ (in contrast with USD 380 from the previous change) but low returns. In this case, farmers should consider if this performance is acceptable and worth the risk, i.e., changes in input/output prices, risk of N losses at high N levels etc. Lemaire et al*.*^7^ call attention, for example, to the losses of N by volatilization of ammonia (NH_3_) and by the emission of nitrous oxide (N_2_O) at high N fertilization levels.

Also, demand for stocker increases after Nitrogen fertilization and farmers must consider the availability of their own funds to purchase more animals or their conditions to access rural credit before commit to any particular fertilization rate. Finally, it is worth noting that the higher the input and technology levels, the higher is the need for sound management practices, which are often an important constraint for Brazilian beef farmers.

## Conclusion

Increasing N fertilization rates from 100 to 300 kg ha^−1^ in *Panicum maximum* Mombaça guinea grass pastures improves forage accumulation rate, stocking rate, nutritive value, body weight gain, and animal and economic performance per area. However, the efficiency of N use (body weight gain per additional kg of N applied) decreases, while the marginal costs increase as N doses increase. Thus, the Mombaça guinea grass pasture fertilized with 200 kg ha^−1^ significantly increases the net return (US$ 380 ha^−1^) and presents the cost–benefit ratio, remaining the best economic alternative even when there is a 25% reduction in productivity (scenario pessimistic). In this sense, it is suggested that N fertilization for Mombaça guinea grass pasture should be between 200 and 300 kg ha^−1^.

Therefore, the establishment of sustainable intensification of Mombaça guinea grass pastures in the Brazilian Cerrado with annual N fertilization associated with adequate grazing management is biologically and economically viable.

## Materials and methods

### Ethics statement

All methods were carried out in accordance with the guidelines established in Normative Resolution no. 25, of September 29, 2015, of the National Council for the Control of Animal Experiments. The Ethics Committee on the Use of Beef Livestock Animals at EMBRAPA—CEUA / EMBRAPA, under licensing nº 003/2015 and 009/2016, approved all experimental protocols. Study is reported in accordance with The ARRIVE (Animal Research: Reporting of in vivo Experiments) guidelines^47^.

### Site, treatments, and experimental design

The experiment was conducted at the National Beef Cattle Research Centre, Campo Grande, MS, Brazil (latitude 20°27′S, longitude 54°37′W, and 530 m altitude), from October 2014 to September 2017. According to Köppen’s classification, the climate is characterized as a rainy tropical savanna, subtype Aw, with seasonal rainfall distribution and dry winter period, generally from May to September. Precipitation and temperature data during the experimental period (Fig. 4) were collected from a weather station, located within 3 km from the experimental site. Soil water balance (Fig. 5) was calculated based on the method described by Thornthwaite and Mather^48^.

The grazing experiment was conducted in well-established Mombasa guineagrass pastures, established in 2008. Soil at the experimental site were classified as Dystrophic Red Latosol^49^, with 30% clay. Initial and final soil samples (May 2014 and 2017) were collected from 0–10 cm and 0–20 cm depth and analyzed for chemical composition (Table 1). These results were utilized as a baseline for fertilization, and in October of each grazing year, pastures were fertilized with 35 kg P ha^−1^ and 66 kg K ha^−1^.

The experiment was laid out in a randomized complete block design, with three treatments and three replicates, for a total nine-pastures each measuring 1.5 ha (module). Treatments were three N-rates: 100, 200 and 300 kg N ha^−1^, henceforth referred as N100, N200 and N300, respectively. Nitrogen fertilizer was applied as urea equally distributed, divided into four applications given in October, December, January, and February.

Each pasture was divided with a conventional fence into six paddocks of 0.25 ha. Rotational stocking was implemented throughout the rainy period, with rest periods determined based on target pre-grazing canopy heights between 80 and 90 cm. Pre-grazing canopy height targets were chosen based on previous research, considering a canopy light interception between 90 and 95%^50^. During the dry period, due to low herbage accumulation, normally recorded for Mombasa guineagrass pasture in this period^9^, it was decided to reduce target pre-grazing canopy height to 60 to 70 cm. A post-grazing height of 45 cm was maintained for all treatments.

Throughout the study, canopy height was measured twice per week using a 1-m ruler, through systematic readings performed along five transect lines (eight measurement points per transect) in all paddocks. These canopy height readings were taken from ground level to the ‘leaf horizon’ on the top of the canopy as a reference. The post-grazing heights were measured as soon as the cattle were removed from the paddock.

In October 2014, fifty four steers (approximately 10-mo old and initial average body weight of 285 ± 14 kg) were randomly assigned to the experimental units (pastures); the differences in allocation weight across treatments were not significant (*P* > 0.05) at the beginning of the growing season. The groups of six steers remained in the same pasture for one rainy season as tester animals. Sixty steers were kept at a reserve pasture (9.0 ha Massai guinea grass) and used whenever necessary to maintain canopy height targets in a put-and-take management system. Stocking rate was adjusted twice a week.

During the dry season, the number of tester steers was reduced to three animal per pasture, due to the small dimensions of the pastures (1.5 ha) relative to the body weight of steers. However, there were times when the pastures, regardless of treatment or block, had to be destocked and the test animals were placed in reserve pastures. The number of days without grazing was recorded (Table 2). In these cases, the animal performance was not accounted for.

In October 2015 and 2016, the steers were replaced with other animals of the same category. The breeds used were: ½ Senepol × ½ Caracu, during the first and second years; and ½ Brahman × ½ Angus in the third year.

### Herbage measures

The pre- and post-grazing forage mass, its morphological composition and the forage accumulation rate were estimated in the six paddocks of each pasture, in each grazing cycle. Pre- and post-grazing forage masses were estimated by cutting nine randomly selected quadrants (1 × 1 m) at ground level. The pre- and post-grazing forage masses were estimated by cutting nine randomly selected quadrats (1 × 1 m) at the soil level.

The samples were divided into two sub-samples: one was weighed and dried in forced-air drying oven 65 °C until constant weight was achieved and weighed again to estimate forage mass. The other sample was separated into leaf (leaf blade), stem (sheath and stem), and dead material; the percentage of the total dry weight for each component was determined, and the leaf:stem ratio calculated. The herbage accumulation rate was calculated as the difference between the current pre-grazing and the preceding post-grazing forage mass, considering only the green portion (leaves and stems) divided by the number of days between samples.

Leaf blade samples were ground to 1 mm and analyzed for concentrations of crude protein (CP), neutral detergent fiber (NDF) and acid detergent lignin (ADL) concentrations, and in vitro digestible organic matter (IVDOM), using the near-infrared reflectance spectrophotometer (NIRS) system^51^.

### Animal responses

All steers were weighed every 28-d, following a 16-h period fasten period. The body weight gain of tester steers was used to calculate ADG. The stocking rate per cycle was calculated according to Petersen and Lucas^52^ as the sum of animals per day (tester and shepherd steers) that remained in each of the six paddocks (0.25 ha) divided by the duration of the grazing cycle (days). The obtained value was divided by the pasture area (1.5 ha). The stocking rate it was expressed in animal unit equivalents (450 kg body weight) per hectare. The BWGA (ha) was calculated by multiplying the ADG of the tester steers by the number of steers (testers and grazers) retained per module and per grazing cycle^52^.

### Statistical analysis

The data were grouped according to the season, where dry season was considered from May to September, and rainy season from October to April. Pasture was considered the experimental unit for both herbage and animal responses. The response variables were analyzed using the method of least squares, using the GLM procedure from SAS (Statistical Analysis System, version 9.4), considering the block, season, experimental year as class variables and their interactions and the N rates as continuous variable. Based on previous analyses, the highest-order polynomial was selected. When *p* > 0.05 or r^2^ < 0.60, the response variables were analyzed by fitting mixed-effects models using the MIXED procedure in SAS. The Akaike information criterion was used to select the covariance matrix^53^. The applied model included the random effect of the blocks and the fixed effects of the N rate, experimental year, season of the year, and their interactions. Year was considered fixed effect because the year effects and interactions with year were of interest due to different rainfall patterns (Figs. 4 and 5). The means were estimated using LSMEANS statement. Tukey test was utilized for mean comparisons, and significance declared at 5%.

### Economic analysis

Given the biological results, economic analysis followed accordingly. With this purpose, a partial budget analysis was undertaken. According to Soha^43^, partial budgeting is a planning tool and a decision framework helpful to assess costs and benefits of a specific change in a farm (for example, fertilization levels, as proposed here) in comparison to the current situation. The author claims that “it focuses only on the changes in income and expenses that would result from implementing a specific alternative… [and thus] all aspects of farm profits that are unchanged by the decision can be safely ignored”^42^. The conceptual basis of partial budgeting can be found in Barnard and Nix^54^ and in Olson^42^. We calculated the Benefit–Cost Ratio (BCR), which demonstrates the relationship between the relative costs and benefits of a project or proposed change. The average market prices of 2019 were used for all inputs, labor and projected receipts to assess the economic performance of N100, N200 and N300 treatments. For the enterprise budget analysis, we considered both fixed and variable costs. The fixed costs comprised cattle purchase (1,276.22 USD ha^−1^ for three weaned calves of 285 kg LW) and pasture establishment costs, involving soil preparation and conditioning (USD 94.26 ha^−1^), the initial fertilization (USD 346.05 ha^−1^) with nitrogen, phosphorous, potassium (NPK formulae) and micronutrients, and labor (USD 38.74 ha^−1^). Given the establishment of new perennial pasture provides benefits throughout its lifetime, usually more than a year, it was necessary to convert the associated costs (USD 479.04) to a year-based cost. We did this by dividing this amount by three, which corresponds to the experimental years, of which we had the experimental data at hand. The alternative of using an investment analysis was discarded because it would require additional data to build a cash flow spanning the pasture total lifetime (which is quite arbitrary and reliant on its management) and simulation data to estimate the beef production over this period, introducing some bias to the analysis. Model simulation, therefore, was out of the scope of this study.

The variable costs included maintenance fertilization (i.e. levels of N fertilizer and labor) and cattle management (e.g. minerals, vaccines, labor and vet supplies), proportionally to stocking rates allowed for by the N rates. Income was estimated for each treatment as a result of the average yield (kg of body weight. ha^−1^) multiplied by the average beef price in 2019 (1.39 USD kg^−1^ LW). The partial budget considered the pairs of consecutive treatments for comparison (i.e., N100 versus N200 and N200 versus N300), following the guidelines of Soha^43^, since farmers are more likely to make incremental changes rather than radical changes. To assess the treatments economic viability, we followed the rules: the greater the increase in net profit and BCR, the more profitable the level of N fertilization is; additionally, any level of N fertilization should only be recommended if BCR > 1.0.

An alternative scenario was also considered for analysis, where beef production reduced 25% across treatments, to represent possible lags between experimental and commercial conditions. As suggested by Olson^42^, the simulation of scenarios is helpful to understand beforehand the potential benefits and drawbacks of future interventions or plans.

## Data Availability

All data generated or analyzed during this study are included in this published article.
